# Scrotal temperature change during running in naked humans

**DOI:** 10.1038/s41598-025-21699-8

**Published:** 2025-10-29

**Authors:** Yasuho Takii, Makoto Fukuda, Kazuyuki Kanosue, Tamae Yoda

**Affiliations:** 1 Institute of Sport Science, ASICS Corporation, Kobe, 651-2271 Hyogo Japan; 2 Faculty of Business Administration, Reitaku University, Kashiwa, Chiba Japan; 3 Institute of Health and Sports Science and Medicine, Juntendo University, Inzai, Chiba Japan; 4 Faculty of Sport Sciences, Waseda University, Tokorozawa, Saitama Japan; 5 Faculty of International Liberal Arts, Dokkyo University, Soka, Saitama Japan

**Keywords:** Human, Male, Exercise, Running, Thermoregulation, Scrotum, Physiology, Testis

## Abstract

Male reproductive organs are functionally affected by high body temperature. This study aimed to clarify the thermoregulation mechanisms of the human scrotum during running, and experiments were conducted to investigate temperature changes in the whole body and the scrotum. Eleven male long-distance runners performed treadmill running at 60%$\:{\dot{\text{V}}}_{{\text{O}}_{\text{2max}}}$ for 60 min in an indoor environment at 27 °C and 40% relative humidity. Immediately after the start of the run, the skin temperatures in areas other than the scrotum decreased or showed a tendency to decrease. In contrast, the scrotal temperature increased immediately after the start of the run. After the end of the run, the scrotal temperature decreased, whereas the skin temperatures in other body parts increased. An increase in scrotal temperature immediately after the start of the run was observed even in the situation where the scrotum was not affected by heating due to contact with the thighs. A possible explanation for this phenomenon is that the scrotum may have been elevated with the contraction of the dartos muscle and the cremaster muscle as a result of increased sympathetic nerve activity. Additionally, it cannot be ruled out that a specific vasodilation mechanism could play a role in this occurrence.

## Introduction

Excessive heat stress has been reported to have detrimental effects on cognitive and physical performance, as well as overall health, through mechanisms such as excessive increases in core body temperature, elevated heart rate, and dehydration^1,2^. To avoid these adverse effects, humans utilize various mechanisms, including behavioral, autonomic, and endocrine thermoregulation, to mitigate the harmful consequences of heat stress^3,4^. In many mammalian species, the temperature of the testes and scrotum is observed to be 2–6 °C lower than the core body temperature^5^. Heat is a factor that reduces the probability of success in mammalian reproduction. For example, sperm motility decreases when the temperature of the scrotum increases^6^. High temperatures in the testes have been observed to induce the apoptosis of germ cells directly or indirectly via a decrease in testosterone concentration^7–10^. Furthermore, low testicular temperature has been shown to increase the amount of sperm stored in the epididymis^11^.

In terms of thermoregulation, the body is divided into two sections: the shell and the core. The shell is the external layer, including the skin, the temperature of which fluctuates significantly depending on the thermal condition of the environment. In contrast, the core refers to the internal layer, including the central nervous system and internal organs, the temperature of which is relatively stable^12^. The skin is further divided into glabrous skin and non-glabrous skin, which have different morphological characteristics and respond differently to thermal stimulation. Glabrous skin is limited to the distal parts of the body, such as the hands, feet, and parts of the face, and has the common characteristics of being hairless, having a dense network of blood vessels, arteriovenous anastomoses, and a large surface-area-to-volume ratio. It contributes to thermoregulation by exploring the environment and efficiently transferring heat to or from the surrounding environment^13,14^. It has also been reported that blood flow in glabrous skin is regulated only by the adrenergic vasoconstrictor system^15^. On the other hand, non-glabrous skin covers a more proximal area than glabrous skin and is characterized by the absence of arteriovenous anastomosis and the presence of hair follicles^13,14^. It produces important input signals for the thermoregulation owing to its large surface area. In addition, while glabrous skin functions as a radiator, non-glabrous skin also functions as an insulator^13^. Furthermore, blood flow in the non-glabrous skin is controlled by both the adrenergic vasoconstrictor system and non-adrenergic vasodilator system, unlike the glabrous area^15^.

The scrotum has five specific anatomical features that maintain a lower temperature relative to the trunk. These include the dartos muscle, the cremaster muscle, the counter-current heat exchange system in the testicular vascular supply, the absence of a subcutaneous fat layer in the skin, and the presence of abundant sweat glands in the skin^16,17^. The dartos muscle is a smooth muscle located inside the scrotum that controls the scrotal skin dynamics^16^. Relaxation of the dartos muscle promotes heat loss from the scrotal skin and contributes to testicular cooling by distancing the scrotum from the heat source of the trunk^5^ and facilitating dilation of blood vessels that run between the dartos muscles^18^. On the other hand, contractions of the dartos muscle are promoted when the ambient temperature drops, and the testes are brought closer to the heat source of the body trunk at the same time as the blood flow to the skin surface decreases^5^. The cremaster muscle is a skeletal muscle found in the spermatic cord, and its contraction and relaxation control the lifting and lowering of the testes^5,19^. The testicular artery in the spermatic cord carries blood from the abdominal aorta to the testis, and this artery descends through the pampiniform plexus of the veins that returns blood from the testis. This structure acts as a field of heat exchange between the artery and the surrounding veins, thereby maintaining a low scrotal temperature. This heat exchange results in cooler blood flow in the testes than in other organs in the body^20^. Subcutaneous fat generally has an insulating effect, reducing heat loss from the body to the environment. The absence of subcutaneous fat in scrotal skin promotes heat loss in testes^16^. In addition, sweating of the scrotal skin, due to the presence of numerous sweat glands, helps to keep the temperature of the testes lower than that of the body core^21^.

As mentioned above, a high body temperature is thought to impair the reproductive function in male animals, and the testes and scrotum have mechanisms to prevent this. However, in humans, the scrotum is typically covered by clothing, and owing to limited research on measurements taken without clothing, whether or not humans possess specific thermoregulatory functions for the scrotum remains unclear. Furthermore, most studies on the effects of heat on the human scrotum and testes have focused on the effects of ambient temperature^22^, posture^6,23^, clothing^6,24^, and direct heating^23,25^ or cooling^26–28^ of the scrotum on sperm count and sperm quality. These studies have investigated the effects of external thermal factors on the body as a whole or on the scrotum. In contrast, exercise is an activity in which the body actively generates heat and this situation causes high heat stress; however, only a few studies have so far focused on the relationship between exercise and the scrotal temperature, in which the scrotal temperature was observed to be low during running in comparison to that of a normal male sitting for 60 min with his thighs together^6^. A recent systematic review discussed the impact of endurance exercise and training, including running, on the semen quality, suggesting that there may be some influence, although definitive conclusions have not yet been reached^29^. Moreover, there have been many studies on body temperature changes and temperature distribution during exercise in humans; however, these studies have not focused on the scrotum^30–32^. Furthermore, although various interventions to alleviate heat stress in high-temperature environments have been investigated, most have focused on cooling the head or upper body^33–35^, with little attention given to the lower body, including the scrotum. Consequently, the necessity and effectiveness of cooling interventions for the scrotum under high heat stress has not been sufficiently examined.

Therefore, this study assessed the mechanism of thermoregulation of the human scrotum during exercise, where local heat stress is particularly significant, as clothing is typically necessary. Specifically, we evaluated scrotal temperature changes when humans exercised without clothing to eliminate the influence of attire. In addition, because it has been suggested that contact of the scrotum with the thighs could affect the temperature of the scrotum in humans^23^, the effect of running on scrotal temperature was also analyzed under conditions in which the scrotum was not passively heated by heat conduction from the thighs.

## Methods

### Experiment 1

#### Subjects

Eleven male student athletes (age, 21.1 ± 1.7 years; height, 173.0 ± 4.5 cm; weight, 56.9 ± 3.4 kg [mean ± standard deviation (SD)]) from a university track and field team in Japan participated in the study. The subjects were mainly athletes who competed in middle-distance (800 m) or long-distance (5,000 m and 10,000 m) running. This study was performed in compliance with the declaration of Helsinki and approval was obtained from the Research Ethics Committee of ASICS Corporation. The purpose and content of the study and possible risks were explained to each subject, and written consent was obtained before the experiments were conducted. The Covid-19 infection prevention measures were approved by the Risk Management Committee of ASICS Corporation, and both the subjects and the experimenter took various infection prevention measures before conducting the experiments. Subjects were requested to avoid excessive alcohol consumption and high-intensity exercise the day before and the morning of the experiment. The experiment was conducted in a climate chamber (Ohnishi Netsugaku Co., Ltd., Osaka, Japan) with a set temperature of 27.5 °C and a relative humidity of 40%. All subjects were instructed to arrive at the laboratory at 10 a.m. on the day of the measurement. The subjects spent approximately 1.5 h at rest in a standing position, while all measurement devices were attached.

#### $\:{\dot{\text{V}}}_{{\text{O}}_{\text{2max}}}$ test

First, maximal oxygen uptake ($\:{\dot{\text{V}}}_{{\text{O}}_{\text{2max}}}$) was measured in each subject^36^. The subjects performed an incremental test on a treadmill (LodeValiant VS-XT2, Lode BV, Groningen, Netherlands) placed in a climate chamber from 7 to 23 days prior to the main experiment. The mean (± SD) ambient temperature and relative humidity during the test were 18.4 ± 0.1 °C and 47.6 ± 2.4%, respectively. Oxygen uptake ($\:{\dot{\text{V}}}_{{\text{O}}_{\text{2}}}$) was measured on a breath-by-breath basis using a metabolic gas analyzer (AE-310, Minato Medical Science, Osaka, Japan). Heart rate (HR) was measured using a wristwatch heart rate monitor (Pacer Pro, Polar Electro Oy, Kempele, Finland). The treadmill speed was set from 10 km/h to a maximum of 18 km/h, and was increased by 2 km/h every 4 min. The treadmill inclination was 1% throughout the study period. Measurements were terminated at the speed at which the subject reached 80% of the predicted maximum heart rate obtained by subtracting his age from 220 (0% as the resting heart rate), or at the speed at which he judged that he could not maintain this speed. $\:{\dot{\text{V}}}_{{\text{O}}_{\text{2max}}}$ was estimated from the relationship between $\:{\dot{\text{V}}}_{{\text{O}}_{\text{2}}}$ and HR at the end of the test, and running speed corresponding to 60%$\:{\dot{\text{V}}}_{{\text{O}}_{\text{2max}}}$ was calculated for each subject^36^.

#### Exercise protocol

The exercise task consisted of 60 min of treadmill running at 60%$\:{\dot{\text{V}}}_{{\text{O}}_{\text{2max}}}$. Resting in a static standing posture was performed for 30 min before and 10 min after the exercise task. Subjects who were unable to maintain a standing position due to fatigue immediately after the end of the run were excused from the study without a period of standing rest. The temperature and humidity in the climate chamber during the experiment were measured using a wet-bulb globe temperature monitor (WBGT-203B; Kyoto Electronics Manufacturing Co., Ltd., Kyoto, Japan). The temperature and relative humidity were measured every 5 min and the average values of the temperature and relative humidity were calculated during the period when the subjects were in the room, including during experiment preparation. The mean (± SD) room temperature and relative humidity during the experimental period were 27.5 ± 0.1 °C and 41.7 ± 0.2%, respectively. The subjects held the treadmill handrail with their hands and maintained a static standing posture with their legs spread wider than their shoulders. The examiner visually confirmed that there was no contact between the scrotum and medial side of the thigh. The subjects wore no clothing except for ankle-length short socks on the feet and a pair of running shoes for road running distributed for the experiment while standing still and running.

#### Measures

The skin temperature, core temperature, HR, ventilatory and circulatory responses, sweat rate, and thermal sensation of each body part were measured during 30 min of static standing before running and during 60 min of treadmill running. Six of the 11 subjects (age: 21.5 ± 1.6 years, height: 172.8 ± 5.9 cm, weight: 56.4 ± 3.2) who were able to continue the experiment without the influence of fatigue after running continued the measurements except for subjective thermal sensation measurements during the 10 min of static standing after running.

#### Skin temperature

**Fig. 1:**
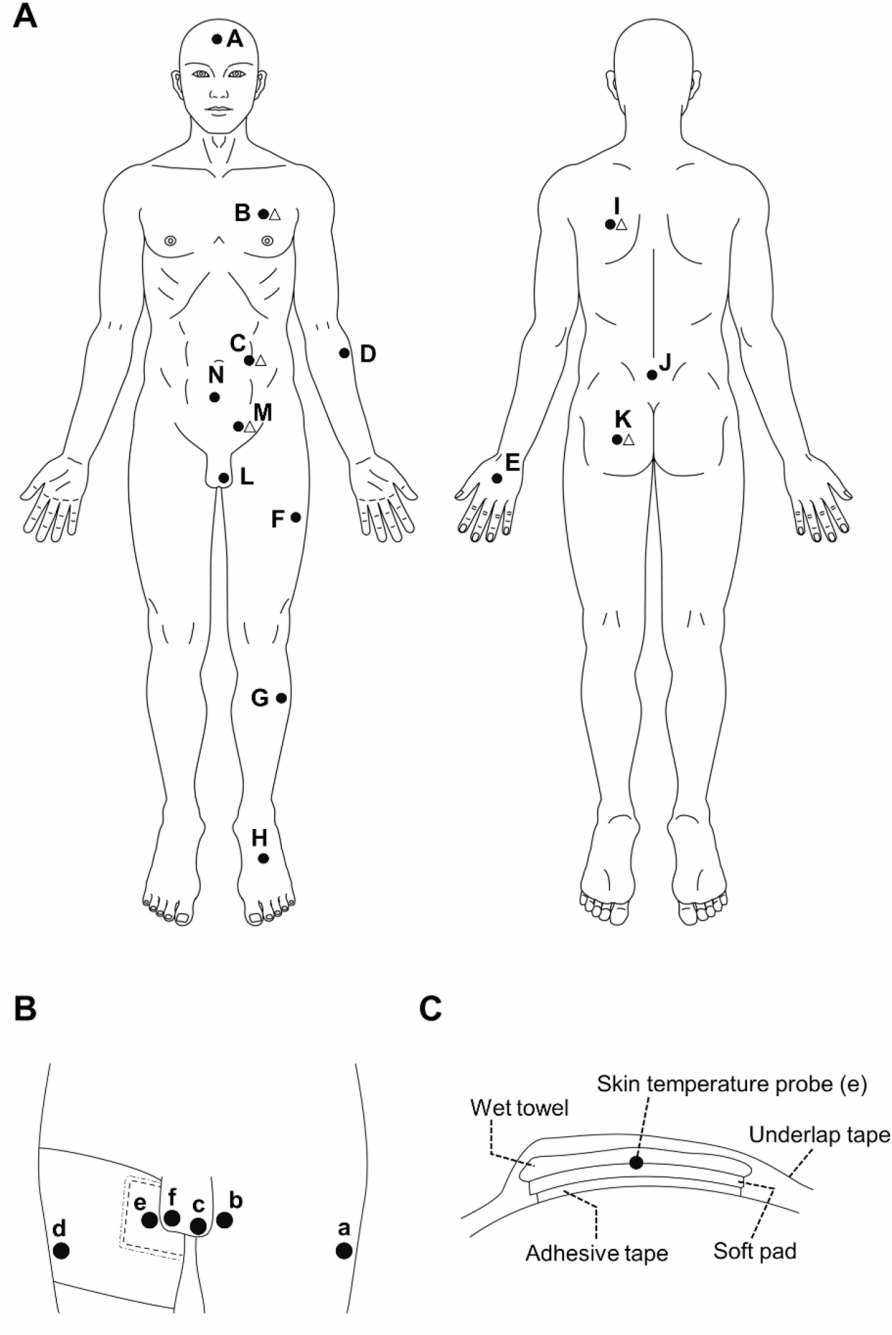
(**A**) Points of skin temperature (●) and sweat rate measurement (△) in Experiment 1; A: forehead; B: chest; C: abdomen; D: forearm; E: hand; F: thigh; G: shank; H: foot; I: back; J: upper buttocks; K: buttocks; L: scrotum; M: groin; N: lower abdomen. (**B**) Points of skin temperature measurement in Experiment 2; a: left lateral thigh; b: left medial thigh; c: left scrotum; d: right lateral thigh; e: right medial thigh; f: right scrotum. (**C**) A cross-sectional view around the sensor on the right medial thigh in Experiment 2.

Data loggers (N543, Nikkiso-Therm Co., Ltd., Musashino, Japan) were used to measure the skin temperature. Skin temperature probes (ITP082-25, Nikkiso-Therm Co., Ltd., Musashino, Japan) were attached to 14 locations on the center of the forehead, lower abdomen, upper buttocks and left side of the back, chest, abdomen, groin, buttocks, forearm, hand, thigh, shank, and scrotum (Fig. 1A), which were attached to the skin using medical tape. The temperatures were recorded every 10 s, and the average values were calculated every 1 min.

#### Core temperature

The core temperature was measured using a data logger and core temperature probe (ITP010-11, Nikkiso-Therm Co., Ltd., Musashino, Japan). A styrofoam ball (diameter, 1 cm) was attached 17 cm from the tip of the probe to prevent it from falling out while the subject was running and then covered with a disposable rubber cover. The participants inserted the tip of the probe, including the ball, into their bodies through their anus. The temperature was recorded every 10 s and the average value was calculated every 1 min. In the measurements of 3 of the 11 subjects, the probe was found to have fallen out during running. The probe was reinserted within 6 min after the subject stopped running.

#### Ventilatory and circulatory responses

Using a metabolic gas analyzer, the following were measured as ventilatory and circulatory response indicators on a breath-by-breath basis: oxygen uptake ($\:{\dot{\text{V}}}_{{\text{O}}_{\text{2}}}$), carbon dioxide output ($\:{\dot{\text{V}}}_{{\text{CO}}_{\text{2}}}$), gas exchange ratio (R), respiratory rate (RR), minute ventilation (V_E_), expiratory tidal volume (TV_E_), inspiratory tidal volume (TV_I_), and ventilatory equivalent for carbon dioxide (V_E_/$\:{\dot{\text{V}}}_{{\text{CO}}_{\text{2}}}$) and the average value every 1 min was calculated.

#### HR

A HR monitor (H10 N, Polar Electro Oy, Kempele, Finland) was attached to the chest of the subject using a dedicated rubber band. The HR was measured every second, and the average value was calculated every minute.

#### Sweat rate

The sweat rate was measured using a perspiration meter (OKS-08HM and POS-02, ASE Giken Co., Ltd, Aichi, Japan). As shown in Fig. 1A, a perspiration sensor probe (diameter, 1.8 cm) was attached to the skin using double-sided tape at the following points: the central part of the left pectoralis major muscle (chest), the central part of the left trapezius muscle (back), the middle part of the left and right superior iliac spines (lower abdomen), the left groin (groin), and the central part of the left gluteus maximus muscle (buttocks). The sweat rate was recorded every 0.1 s and the average value was calculated every 1 min.

#### Thermal sensation

A touch panel (LCD-MF241FVB-T-A, I-O DATA DEVICE, Inc., Ishikawa, Japan) was fixed to the operation panel of the treadmill using a monitor arm. The subjects were visually presented with a 315 mm Visual Analog Scale (VAS) on the screen, with the left end corresponding to “extremely cold” and the right end corresponding to “extremely hot.” The subjects were also instructed to draw a line across the horizontal axis of the thermal sensation scale for the whole body and the scrotum using a stylus or finger to indicate the position corresponding to their own sensation^37^. Data were recorded every 5 min during rest and the run. Values were quantified with the left end of the VAS set at − 50 points and the right end at 50 points.

#### Data analysis

The mean skin temperature was calculated using Hardy and DuBois’s 7-point method^38^. The %$\:{\dot{\text{V}}}_{{\text{O}}_{\text{2max}}}$ during running was calculated for each subject based on the ratio of $\:{\dot{\text{V}}}_{{\text{O}}_{\text{2}}}$ to $\:{\dot{\text{V}}}_{{\text{O}}_{\text{2max}}}$ during running. The $\:{\dot{\text{V}}}_{{\text{O}}_{\text{2}}}$ value during running was unavailable for one of the 11 subjects in the post-experiment analysis. Therefore, the %$\:{\dot{\text{V}}}_{{\text{O}}_{\text{2max}}}$ was estimated based on the relationship between HR and $\:{\dot{\text{V}}}_{{\text{O}}_{\text{2}}}$ during the $\:{\dot{\text{V}}}_{{\text{O}}_{\text{2max}}}$ test. For subjects with the probe reinserted during running, when the values of %$\:{\dot{\text{V}}}_{{\text{O}}_{\text{2max}}}$ deviated from the average value ± 3SD from 3 to 60 min after the start of the run, the data for skin temperature, core temperature, HR, %$\:{\dot{\text{V}}}_{{\text{O}}_{\text{2max}}}$, and sweat rate during the same interval were excluded from the analysis.

#### Statistical analysis

Values are expressed as the mean ± SD. The data on skin temperature, mean skin temperature, and core temperature during rest and exercise were subjected to a two-way analysis of variance (ANOVA) to analyze the main effects (place, time) and interactions (place × time). Significant ANOVA effects were followed up with Tukey–Kramer tests or paired-samples t-tests as a post hoc analysis. SPSS (IBM SPSS Statistics 26, IBM, NY, USA) was used to perform the statistical analyses. Statistical significance was set at *p* < .05.

### Experiment 2

As one of the factors that cause changes in scrotal skin temperature immediately after starting the run, there is a possibility of heat conduction due to contact between the scrotum and thigh^23^ through the change in posture from static standing to running. The following experiments were conducted to examine this effect.

#### Subjects

Six male student athletes (age, 21.3 ± 1.2 years; height, 173.5 ± 5.4 cm; weight, 59.1 ± 5.2 kg [mean ± SD]) from a university track and field team in Japan participated in the study. The subjects were mainly athletes who competed in middle-distance (800 m) or long-distance (5,000 m and 10,000 m) running. Participation followed the same procedure for each subject as in Experiment 1. There was a mixture of participants who participated in Experiment 1 and those who did not. For those who did not participate in Experiment 1, $\:{\dot{\text{V}}}_{{\text{O}}_{\text{2max}}}$ was estimated using the same method as in Experiment 1. The mean (± SD) ambient temperature and relative humidity during the $\:{\dot{\text{V}}}_{{\text{O}}_{\text{2max}}}$ test were 18.4 ± 0.1 °C and 47.6 ± 2.4%, respectively. Subjects were requested to avoid excessive alcohol consumption and high-intensity exercise the day before and the morning of the experiment. The experiment was conducted in a climate chamber with a set temperature of 27.5 °C and a relative humidity of 40%. All subjects were instructed to arrive at the laboratory at 10 a.m. on the day of the measurement. The subjects spent approximately 1 h at rest in a standing position, while all measurement devices were attached.

#### Exercise protocol

The exercise task consisted of 5 min of treadmill running at 60%$\:{\dot{\text{V}}}_{{\text{O}}_{\text{2max}}}$. The participants rested in a static posture for 15 min before the exercise task. The temperature and humidity in the climate chamber during the experiment were measured and calculated as described for the Experiment 1. The mean (± SD) room temperature and relative humidity during the experimental period were 27.5 ± 0.2 °C and 41.4 ± 0.3%, respectively. As in Experiment 1, the subjects held the handrails on the side of the treadmill with their hands in a static position with their legs spread wider than shoulder width apart. The examiner visually confirmed that there was no contact between the scrotum and medial side of the thigh. The clothing conditions for the subjects during the experiment were the same as in Experiment 1.

#### Measures

Skin temperature, core temperature, HR, and ventilatory and circulatory responses were measured during 15 min of static standing before running and during 5 min of treadmill running, respectively. Ventilatory and circulatory responses and HR were measured and calculated as described in Experiment 1.

#### Skin temperature

Data loggers were used to measure the skin temperature. Skin temperature probes were attached to 6 locations on the right and left lateral thighs, which were the measurement points of the thigh in Experiment 1, medial thighs, and the bottom of the scrotum using medical tape (Fig. 1B). To prevent the scrotum from being passively heated by heat conduction from the thigh, a soft pad of EVA Foam (Ethylen-Vinyl Acetate Foam; thickness, 5 mm) was attached to the medial side of the right thigh using adhesive tape and medical tape, and the probe was attached to its surface. During the 15-min period of static standing before the run, a towel wet with room temperature water (10–15 °C) was fixed on top of the pad and probe using an underlap to cool the surface of the pad and probe (Fig. 1C). The examiner confirmed that the surface temperature of the pad was lower than that of the scrotum on the same side during static standing. The subject removed the towel immediately before the start of the run. The temperatures were recorded every 10 s, and the average values were calculated every 1 min.

#### Core temperature

The core temperature was measured using a data logger and a core temperature probe. The subjects inserted the tip of the probe, which was covered with a disposable rubber cover, into their body through their anus up to 17 cm from the tip of the probe. The temperature was recorded every 10 s and the average value was calculated every 1 min.

#### Data analysis

The %$\:{\dot{\text{V}}}_{{\text{O}}_{\text{2max}}}$ during running was calculated for each subject based on the ratio of $\:{\dot{\text{V}}}_{{\text{O}}_{\text{2}}}$ to $\:{\dot{\text{V}}}_{{\text{O}}_{\text{2max}}}$ during running.

#### Statistical analysis

Values are expressed as the mean ± SD. The data on skin temperature, mean skin temperature, and core temperature during rest and running were subjected to a two-way ANOVA for the main effects (place, time) and interactions (place × time). Significant ANOVA effects were followed up with a Tukey–Kramer post hoc analysis. SPSS was used to perform the statistical analyses. Statistical significance was set at *p* < .05.

## Results

### Experiment 1

#### Skin and core temperatures

**Fig. 2:**
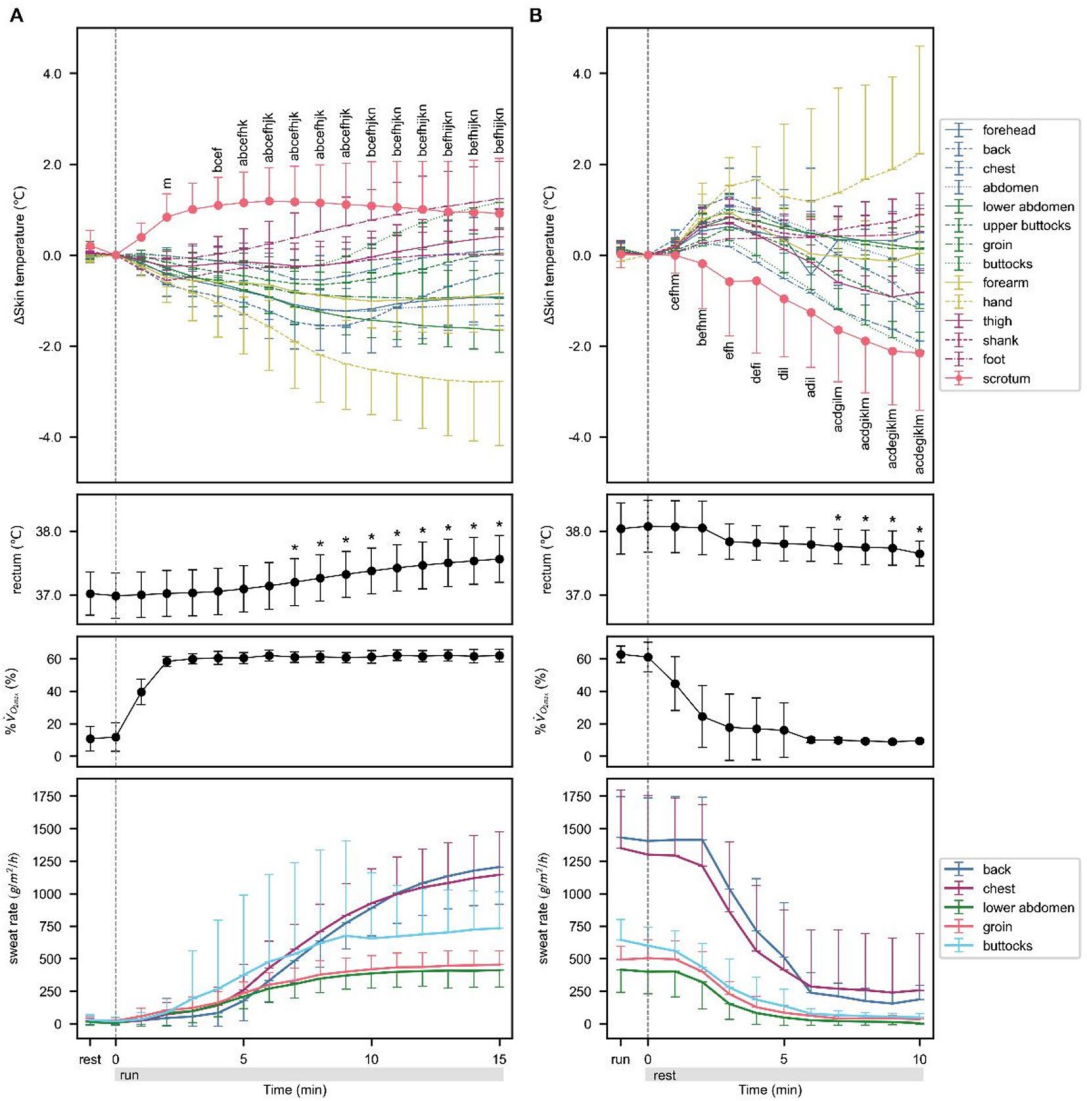
Responses of body temperatures, oxygen uptake and sweating around starting (**A**) and finishing (**B**) running. Values are mean ± SD; *n* = 8–11 (**A**), *n* = 4–6 (**B**). ^a^Significant difference versus 0 min in scrotum(*p* < .05), ^b^Significant difference versus 0 min in forehead (*p* < .05), ^c^Significant difference versus 0 min in back (*p* < .05), ^d^Significant difference versus 0 min in chest (*p* < .05), ^e^Significant difference versus 0 min in abdomen (*p* < .05), ^f^Significant difference versus 0 min in lower abdomen (*p* < .05), ^g^Significant difference versus 0 min in upper buttocks (*p* < .05), ^h^Significant difference versus 0 min in groin (*p* < .05), ^i^Significant difference versus 0 min in buttocks (*p* < .05), ^j^Significant difference versus 0 min in forearm (*p* < .05), ^k^Significant difference versus 0 min in hand (*p* < .05), ^l^Significant difference versus 0 min in thigh (*p* < .05), ^m^Significant difference versus 0 min in shank (*p* < .05), ^n^Significant difference versus 0 min in foot (*p* < .05), *Significant difference versus 0 min in rectum (*p* < .05).

The time-series data for skin temperature, mean skin temperature, core temperature, %$\:{\dot{\text{V}}}_{{\text{O}}_{\text{2max}}}$ and sweat rate from 10 min before to 15 min after the start of the run are shown in Fig. 2A. The skin temperature was expressed as the change from the value at the start of the run, and the core temperature, %$\:{\dot{\text{V}}}_{{\text{O}}_{\text{2max}}}$ and sweat rate were expressed as measured values. The temperatures at the start of the run, and the maximum and minimum temperatures of each body part for 15 min after the start of the run are listed in Table 1. The skin temperatures of the forehead, back, abdomen, lower abdomen, groin, forearm, and hand showed their maximum values at the start of the run and their minimum values after 6 min of the run. The minimum values for the chest, upper buttocks, buttocks, thigh, shank, and foot were recorded between 2 and 8 min after the start of the run, and the maximum values were recorded after 15 min. The rectal temperature, which was measured as an indicator of core temperature, showed a minimum value at the start of the run and a maximum value after 15 min. The scrotum showed the lowest temperature at the start of the run and the highest temperature 6 min after the start of the run. A two-way ANOVA was conducted for skin temperature and core temperature, with time and place as the two factors, and the main effects of time (*F*_*25, 4160*_ = 52.50, *p* < .001, *η*_*p*_^*2*^ = 0.24) and place (*F*_*15, 4160*_ = 266.84, *p* < .001, *η*_*p*_^*2*^ = 0.49) were significant. The interaction between time and place was significant (*F*_*375, 4160*_ = 10.53, *p* < .001, *η*_*p*_^*2*^ = 0.49). The temperature at each measurement point changed significantly from the start of the run to the time points shown in Fig. 2A (all *p* < .05).

**Table 1:** Statistical description of experimental data on local temperatures before and after starting exercise.

		0 min	0–15 min	
			Max	Min
Temperature (°C)	Forehead	35.16 ± 0.45	35.16 ± 0.44	33.93 ± 0.68
	Back	33.42 ± 1.20	32.17 ± 0.51	30.61 ± 0.66
	Chest	33.48 ± 0.61	33.60 ± 0.94	32.93 ± 0.87
	Abdomen	34.03 ± 0.36	34.02 ± 0.35	32.78 ± 0.43
	Lower abdomen	34.03 ± 0.43	34.02 ± 0.43	32.36 ± 0.44
	Upper buttocks	33.53 ± 0.79	33.56 ± 1.04	32.87 ± 0.79
	Groin	34.61 ± 0.41	34.60 ± 0.41	33.65 ± 0.78
	Buttocks	32.63 ± 0.48	33.78 ± 0.74	32.34 ± 0.79
	Forearm	32.18 ± 0.51	32.29 ± 0.72	31.28 ± 0.85
	Hand	32.30 ± 0.73	32.27 ± 1.68	29.48 ± 1.54
	Thigh	32.27 ± 1.69	34.00 ± 0.76	33.35 ± 0.73
	Shank	33.60 ± 0.61	33.83 ± 0.55	33.26 ± 0.49
	Foot	33.82 ± 0.37	34.65 ± 0.86	33.32 ± 1.11
	Scrotum	32.27 ± 0.97	33.45 ± 0.93	32.26 ± 0.96
	Rectum	36.98 ± 0.36	37.56 ± 0.36	36.98 ± 0.35

Values are mean ± SD; *n* = 11.

The time-series data for skin temperature, core temperature, %$\:{\dot{\text{V}}}_{{\text{O}}_{\text{2max}}}$ and sweat rate from 10 min before to 10 min after the end of the run are shown in Fig. 2B. The skin temperature was expressed as the change from the value at the end of the run, and the core temperature, %$\:{\dot{\text{V}}}_{{\text{O}}_{\text{2max}}}$, and sweat rate were expressed as measured values. The temperatures at the end of the run, and the maximum and minimum temperatures of each body part during the 10 min after the end of the run are listed in Table 2. The maximum temperatures were observed in the back, chest, abdomen, upper buttocks, buttocks, forearm, and thigh, 3 min after the end of the run, and minimum temperatures were observed 9 min after the end of the run. The forehead showed the maximum at 2 min after the end of the run and the minimum at 6 min after the end of the run. The lower abdomen, groin, and shank showed a minimum at the end of the run and a maximum at 3 min after the end of the run. The hand and foot showed a minimum at the end of the run and a maximum at 10 min after the end of the run. The scrotal and rectal temperatures showed their maximum temperatures at the end of the run and their minimum temperatures at 9 min after the end of the run. A two-way ANOVA was conducted for skin temperature and core temperature, with time and place as the two factors, and the main effects of time (*F*_*20, 1392*_ = 25.61, *p* < .001, *η*_*p*_^*2*^ = 0.27) and place (*F*_*15, 1392*_ = 79.64, *p* < .001, *η*_*p*_^*2*^ = 0.46) were significant. The interaction between time and place was also significant (*F*_*300, 1392*_ = 8.92, *p* < .001, *η*_*p*_^*2*^ = 0.66). The temperature at each measurement point changed significantly from the end of the run to the time points shown in Fig. 2B (all *p* < .05).

**Table 2:** Statistical description of experimental data on local temperatures before and after finishing exercise.

		0 min	0–10 min	
			Max	Min
Temperature (°C)	Forehead	34.16 ± 1.20	34.68 ± 0.94	33.49 ± 2.15
	Back	35.06 ± 1.11	33.14 ± 0.73	30.67 ± 0.65
	Chest	33.05 ± 1.13	33.52 ± 0.63	31.32 ± 0.79
	Abdomen	31.81 ± 0.94	33.21 ± 0.65	31.60 ± 0.48
	Lower abdomen	30.93 ± 1.01	32.25 ± 0.51	30.93 ± 1.00
	Upper buttocks	33.42 ± 1.16	33.88 ± 1.32	32.03 ± 1.08
	Groin	32.70 ± 1.36	34.14 ± 1.02	32.69 ± 1.36
	Buttocks	32.96 ± 0.92	33.38 ± 0.94	31.25 ± 0.99
	Forearm	31.67 ± 0.96	33.26 ± 1.42	32.05 ± 1.35
	Hand	32.15 ± 2.03	31.37 ± 1.44	29.76 ± 2.40
	Thigh	29.77 ± 2.41	34.53 ± 0.74	32.94 ± 1.23
	Shank	33.74 ± 0.90	34.25 ± 0.60	33.47 ± 0.64
	Foot	33.48 ± 0.65	35.49 ± 0.95	35.05 ± 1.10
	Scrotum	31.39 ± 2.37	31.38 ± 2.37	29.11 ± 2.10
	Rectum	38.07 ± 0.41	38.07 ± 0.40	37.64 ± 0.19

Values are mean ± SD; *n* = 11.

#### %$\:{\dot{\text{V}}}_{{\text{O}}_{\text{2max}}}$ and HR

The time-series data of %$\:{\dot{\text{V}}}_{{\text{O}}_{\text{2max}}}$ during the experiment are shown in Fig. 2. %$\:{\dot{\text{V}}}_{{\text{O}}_{\text{2max}}}$ increased during the first 2 min after starting the run and then reached a plateau, and the mean ± SD of %$\:{\dot{\text{V}}}_{{\text{O}}_{\text{2max}}}$ for all subjects during the period from 3 min to the end of the run was 61.51 ± 3.49%. HR increased considerably during the first 2 min of running and then gradually increased until the end of the run.

#### Sweat rate

Time series data of sweat rate in the back, chest, lower abdomen, groin, and buttocks from 10 min before to 15 min after the start of the run and from 10 min before to 10 min after the end of the run are shown in Fig. 2. At all measurement points, the minimum value was observed at the start of the run and the maximum value was observed 15 min after the start of the run. Furthermore, the maximum value was observed at 1 min after the end of the run, and the minimum value was observed from 8 to 9 min after the end of the run at all measurement points.

#### Thermal sensation

**Fig. 3:**
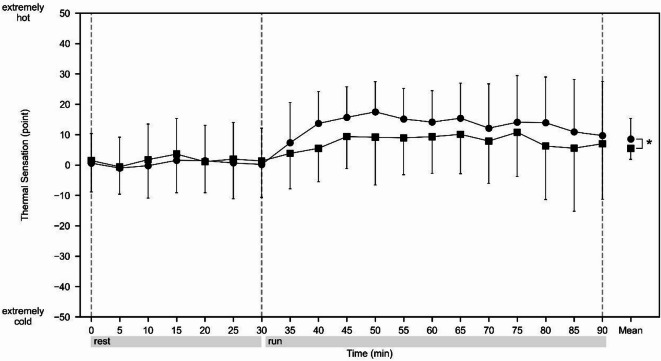
Responses of thermal sensation. Circle (●) shows whole body sensation. Square (■) shows sensation at the scrotum. Values are shown as the mean ± SD; *n* = 11. *Significant difference between whole body sensation and sensation at scrotum for the mean values across all time points (*p* < .05).

The time-series data of the thermal sensation from the start to the end of the experiment are shown in Fig. 3. At the beginning of the run, the mean (± SD) thermal sensation over the whole body and scrotum was 0.18 ± 10.85 points and 1.32 ± 10.82 points, respectively, indicating that the subjects had experienced almost no sensation of being cold or hot. During the phase from the start of the run to 15 min later, both the whole body and the scrotal sensations showed minimum values at the start of the run and maximum values 15 min after the start of the run. The mean ± SD of the thermal sensation over the whole body and scrotum at the end of the run was 9.68 ± 17.80 points and 7.00 ± 18.29 points, respectively. A two-way ANOVA was conducted with time and place as the two factors, and the main effects of time (*F*_*18, 380*_ = 3.48, *p* < .001, *η*_*p*_^*2*^ *=* 0.14) and place (*F*_*1, 380*_ = 6.01, *p* = .015, *η*_*p*_^*2*^ = 0.02) were significant. The interaction between time and place was not significant (*F*_*18, 380*_ = 0.43, *p* = .98, *η*_*p*_^*2*^ = 0.02). There were no significant differences between the mean values at the beginning of the run and at any point during the run. In contrast, the scrotal thermal sensation was significantly lower than that of the whole body throughout the entire measurement period (*p* < .001).

### Experiment 2

#### Skin and core temperatures

**Fig. 4:**
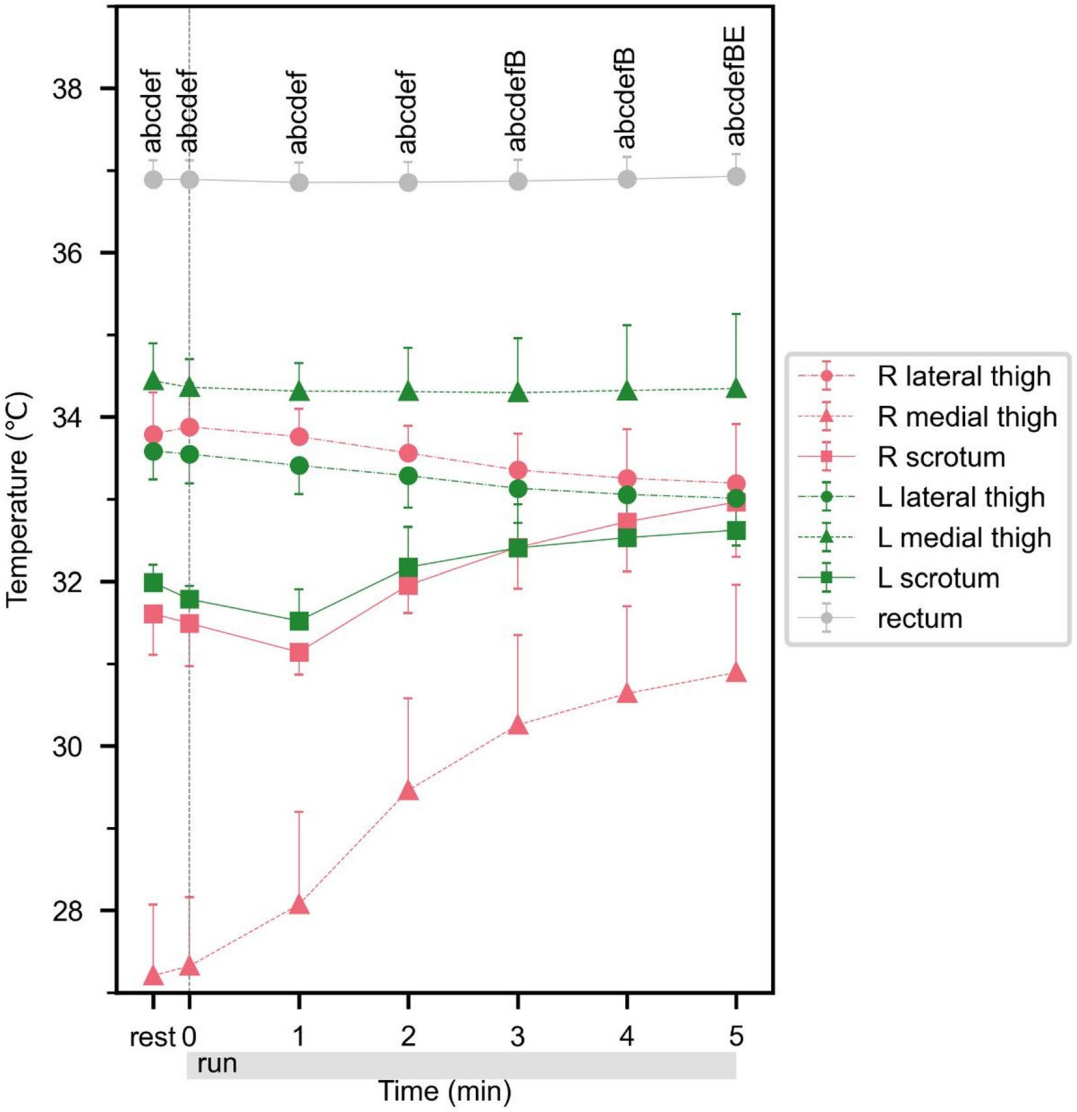
Experiment 2: Responses of skin and core temperatures. Values are mean ± SD; *n* = 6. ^a^Significant difference versus right medial thigh in right lateral thigh (*p* < .05), ^b^Significant difference versus right medial thigh in right scrotum (*p* < .05), ^c^Significant difference versus right medial thigh in left lateral thigh (*p* < .05), ^d^Significant difference versus right medial thigh in left medial thigh (*p* < .05), ^e^Significant difference versus right medial thigh in left scrotum (*p* < .05), ^f^Significant difference versus right medial thigh in rectum (*p* < .05), ^B^Significant difference versus 0 min in right scrotum (*p* < .05), ^E^Significant difference versus 0 min in left scrotum (*p* < .05).

The time-series data for the skin and core temperatures are shown in Fig. 4. The maximum values of the left and right lateral thighs were observed at the start of the run and the minimum values were observed at the end of the run. The mean (± SD) temperature of the left medial thigh was 34.32 ± 0.02 °C throughout the run. The right medial thigh and rectal temperatures showed minimum values at the start of the run and maximum values at the end of the run. The left and right scrotal temperatures showed minimum values 1 min after the start of the run and maximum values at the end of the run. The temperatures at the start of the run, and the maximum and minimum temperatures during the run, are listed in Table 3. A two-way ANOVA was conducted with time and place as the two factors, and the main effects of time (*F*_*6,245*_ = 10.78, *p* < .001, *η*_*p*_^*2*^ = 0.21) and place (*F*_*6, 245*_ = 691.72, *p* < .001, *η*_*p*_^*2*^ = 0.94) were significant. The interaction between time and place was significant (*F*_*36, 245*_ = 7.63, *p* < .001, *η*_*p*_^*2*^ = 0.53). The right medial thigh had significantly lower temperatures than the right scrotum throughout the measurement period (all *p* < .001). Furthermore, the temperature of the right scrotum increased significantly from 3 to 5 min after the start of the run, in comparison to that at the start of the run (all *p* < .05). In addition, the temperature of the left scrotum increased significantly 5 min after the start of the run, in comparison to that at the start of the run (*p* = .039).

**Table 3:** Statistical description of experimental data on local temperatures after starting exercise in experiment 2.

		0 min	0–5 min	
			Max	Min
Temperature [°C]	Right lateral thigh	33.88 ± 0.49	33.87 ± 0.49	33.16 ± 0.68
	Right medial thigh	27.32 ± 0.77	31.01 ± 0.98	27.32 ± 0.76
	Right scrotum	31.49 ± 0.48	33.09 ± 0.64	30.91 ± 0.4
	Left lateral thigh	33.55 ± 0.32	33.54 ± 0.32	32.98 ± 0.53
	Left medial thigh	34.36 ± 0.31	34.35 ± 0.31	34.28 ± 0.26
	Left scrotum	31.78 ± 0.15	32.66 ± 0.57	31.21 ± 0.36
	Rectum	36.89 ± 0.21	36.95 ± 0.24	36.84 ± 0.22

Values are mean ± SD; *n* = 4–6.

#### %$\:{\dot{\text{V}}}_{{\text{O}}_{\text{2max}}}$ and HR

The mean ± SD %$\:{\dot{\text{V}}}_{{\text{O}}_{\text{2max}}}$ and HR during the last 1 min of the 5-min run were 57.45 ± 3.18% and 131.02 ± 15.38 bpm, respectively.

## Discussion

The objective of this study was to investigate the thermal response of the scrotum during running in humans by measuring scrotal temperature in the absence of clothing interference. One of the novel findings of this study was that changes in scrotal skin temperature during the run showed a sharp contrast to those in other parts of the body. While the skin temperature of other parts of the body decreased immediately after the start of the run, the scrotal temperature increased. It is inferred that this unique response to the scrotum would be a result of the combined effects of the thermoregulatory mechanisms common to the non-glabrous skin and those specific to the scrotum, as will be discussed below.

The experiment was conducted in a climate chamber with the temperature and relative humidity set at 27.5 °C and 40%, respectively, and the subjects were not wearing any clothes other than shoes and socks. Skin temperatures, core temperature, and sweat rate were stable during the pre-exercise period of static standing, indicating that almost no sweating had occurred. Hence, thermal equilibrium was probably reached during the rest period in this experimental condition and no thermal stress was provided by the environment. Therefore, it can be said that the present experimental condition was suitable for analyzing the effect of heat produced with exercise on scrotal temperature.

The skin temperatures at rest before the run are shown in Table 1. The values for most of these measurement points were generally similar to those in previous studies that measured the skin temperature during rest under similar temperature and humidity conditions^39,40^. In this study, among the areas where a significant decrease in skin temperature was observed immediately after the start of the run, the forehead, back, and abdomen showed a minimum value 15 min after the start of the run, after which the temperature began to rise. Transient decreases in temperature were also observed in the chest, upper buttocks, buttocks, thighs, and shins, although the differences were not statistically significant. It has been previously reported that, during exercise, skin blood flow first decreases temporarily and then increases with an increase in the core temperature^42,45^. The first decrease and then increase in skin temperature after the start of the run would reflect the change in skin blood flow, which may reflect this mechanism. It should be noted that no area other than the scrotum showed a statistically significant increase in skin temperature as early as 5 min after the start of running.

A notable finding of this study was that the scrotal skin temperature showed a completely opposite change to those of the other parts of the body immediately after the start and end of the run. The temperatures of all parts of the body except the scrotum showed a declining trend between 2 and 3 min after the start of the run. In addition, the skin temperature of all parts of the body except the feet (on which shoes and socks were worn) and the scrotum showed a declining or similar trend until 9 min after the start of the run. However, only in the scrotum did the temperature consistently increase from the start of the run until 15 min after the start of the run, relative to the temperature at the start of the run. This result differs from that reported in a previous study, in which the change in skin temperature in humans wearing boxer shorts during exercise was measured and the scrotal temperature decreased immediately after the start of running^49^. Blood vessels in general skin areas contract at the start of exercise to redistribute blood to actively working muscles, even if it causes partial impairment of thermoregulation^41–44^. Contrary to the general trend, an increased temperature in the scrotum alone may have significant functional importance: namely, it promotes heat dissipation during exercise and protects the testes, which are vulnerable to heat. This function may have been acquired during the process of evolution, when humans originally lived without any clothes or other coverings.

One possible factor causing the change in scrotal skin temperature immediately after the start of the run is heat conduction resulting from contact between the scrotum and thigh due to the postural change from standing to running^23^. Therefore, Experiment 2 was conducted to confirm the validity of the results of Experiment 1. In Experiment 2, a soft pad was attached to the medial thigh which was in contact with the scrotal measurement point, and cooling was applied to the pad in advance to the run. The pad surface temperature was always significantly lower than the scrotal skin surface temperature throughout the measurement period. Therefore, in Experiment 2, even if the scrotum contacted the pad, the scrotal skin surface temperature did not increase simply because of heat conduction. Even under this condition, the scrotal skin temperature increased not only on the left uncooled side but also on the cooled right side after the start of the run (Fig. 4), similarly as observed in Experiment 1. A significant temperature increase was observed from the start of the run on the right scrotal skin temperature after 3 min of the run and on the left scrotal skin temperature after 5 min of the run. Therefore, it is considered that the increase in scrotal skin surface temperature after the start of the run is not due to contact with other parts of the body.

The possible causes of the increase in scrotal skin temperature after the start of the run observed in this experiment include the contraction of the cremaster muscle and dartos muscle. The sympathetic nervous system is activated during exercise, and the blood adrenaline and noradrenaline levels increase^50^. In ram, it has been reported that the dartos muscle contracts in response to noradrenaline via β-adrenergic receptors^51^. In addition, the cremasteric reflex, in which the cremaster muscle contracts in response to the mechanical stimulation of the skin on the upper thigh, might be triggered^52^. At the onset of running, the blood noradrenaline level may have increased, and physical stimulation of the upper thigh could have induced the cremasteric reflex. These responses may have led to elevation of the testicles and scrotum.

Scrotal skin temperature is affected not only by the distance between the scrotum and trunk, but also by vasomotion of the skin blood vessels and sweating. In non-glabrous skin, blood vessels generally constrict immediately after exercise begins due to the activation of the adrenergic vasoconstrictor system. The cholinergic vasodilator system is thought to contribute little to this vasomotor behavior at the start of exercise^41^. If the cutaneous blood vessels of the scrotum are controlled by the adrenergic vasoconstrictor system in the same way as in other parts of the body^41^, the adrenergic vasoconstrictor system would also be activated at the start of exercise. Then, the increase in scrotal temperature could be due to the combined effects of the multiple mechanisms involved in the regulation of testicular and scrotal temperature. However, taking the importance of heat dissipation in order to protect the reproductive organs into consideration, the scrotum might be innervated differently from other parts of the body. The detailed mechanism should be clarified in the future by evaluating the morphology and muscle activity to confirm whether the scrotum is elevated and by evaluating the blood flow on the scrotal skin.

There was no significant change in the thermal sensation of the whole body throughout the measurement period relative to the start of the run. Also, the mean (± SD) thermal sensation of the whole body and scrotum during the measurement period were 8.57 ± 6.76 points and 5.49 ± 3.62 points, respectively, and the subjects felt hot in these areas. In addition, the thermal sensation of the scrotum was significantly lower than that of the whole body (*p* < .001). In Experiment 1, the average skin temperature significantly decreased from 4 to 15 min after the start of the run relative to the start of the run (all *p* < .05), but the thermal sensation did not change significantly during this period. Once the body has reached a steady state after the start of exercise, thermal sensation is affected more by skin temperature or environmental temperature than by core temperature^53^. However, previous studies have shown that thermal sensation is not necessarily correlated with changes in skin temperature immediately after the start of exercise^54,55^, and the results of this study are thought to be similar with these studies. However, the fact that the thermal sensation in the scrotum was significantly lower than that of the whole body was an unexpected result considering the importance of thermoregulation in the scrotum. This suggests that the degree of cooling of the scrotum and testis under the present experimental conditions, i.e. running without wearing any clothes other than shoes and socks, was sufficient to prevent a further increase in the heat sensation. However, how the scrotal thermal condition obtained here influences reproductive functions, such as spermatogenesis, is beyond the scope of the present study because it was not directly assessed. This should be analyzed in future studies, especially in relation to the influence of clothes.

In this study, ethical and experimental difficulties were encountered in measuring the physiological responses of the scrotum during running in human subjects. The ethical concerns included the need to protect participants’ privacy and dignity, as well as to minimize discomfort and potential embarrassment associated with measurements of scrotal elevation, blood flow of the scrotal skin, and local sweat rate, which prompted us to refrain from such measurements. The lack of data on scrotal elevation, blood flow to the scrotal skin, and local sweat rate is a limitation of the present study. Furthermore, although it was not the focus of the present study, the impact of changes in scrotal skin temperature during running on the reproductive function should be addressed in future studies. In addition, investigating the effects of active cooling of the lower body, including the scrotum, on physiological responses to heat stress represents another important area for future research.

In conclusion, during a 60-min treadmill run in the thermoneutral zone with no clothing other than shoes and socks, only scrotal temperature increased immediately after the start of the run, even in the absence of contact-based heating from the thighs, and decreased after the end of the run; the opposite temperature changed to other skin areas. This phenomenon was possibly caused by the elevation of the scrotum due to the contraction of the dartos muscle and the cremaster muscle due to the increased sympathetic activity at the start of the run. However, the mechanism should be investigated in detail in future studies by measuring the blood flow on the scrotal skin surface and testicular elevation during running.

## Data Availability

The datasets generated and/or analyzed during the current study are not publicly available due to the nature of this research, participants of this study did not agree for their data to be shared publicly, but are available from the corresponding author on reasonable request.
